# Increased Neck Tilt/T1 slope ratio may play an important role in patients with cervical kyphosis

**DOI:** 10.1186/s12891-021-04678-8

**Published:** 2021-09-12

**Authors:** Zhibin Lan, Zhiqiang Wu, Yuming Huang, Weihong Xu

**Affiliations:** 1 The Spine Surgery Department, Quanzhou Orthopedic-Traumatological Hospital of Fujian Traditional Chinese Medicine University, Citong Road, Fengze District, 362000 Quanzhou, China; 2 Department of Spine Surgery, The First Affiliated Hospital of Sun Yat-sen University, Guangzhou, China; 3 Department of Spine Surgery, First Affiliated Hospital of Fujian Medical University, 350004 Fuzhou, Fujian China

**Keywords:** Cervical kyphosis, Neck tilt, T1 slope, C2–7 lordosis, NDI

## Abstract

**Background:**

In previous studies, we demonstrated that the T1 slope (T1s) is associated with clinical outcomes, but the results were not specific for individuals. A recent study suggested that an increased pelvic tilt (PT)/sacral slope (SS) ratio may play an important role in the degeneration of lumbar scoliosis and pathogenesis of lumbar spondylolisthesis. Therefore, we aimed to explore the role of neck tilt (NT)/T1s in patients with cervical kyphosis.

**Methods:**

In total, the data of 36 kyphosis patients who underwent anterior cervical hybrid decompression and fusion (ACHDF) for multilevel (3 levels) cervical spondylotic myelopathy were retrospectively analyzed. The radiographic measurements included the T1s, NT, C2–7 Cobb angle, and C2–7 sagittal vertical axis (SVA). The visual analog scale (VAS) and neck disability index (NDI) scores were used to determine the clinical prognosis. Pearson’s correlation coefficient was calculated to assess the relationships among preoperative imaging examination parameters.

**Results:**

The mean C2–7 Cobb angle was − 5.93 ± 3.00° before surgery, 9.67 ± 6.61° after surgery, and 7.91 ± 8.73° at the follow-up. The preoperative NT/T1s ratio was positively correlated with the ΔC2–7 Cobb angle (*r* = 0.358, *p* < 0.05) and negatively correlated with the preoperative C2–7 Cobb angle (*r* = -0.515, *p* < 0.01) and preoperative C2–7 SVA (*r* = -0.461, *p* < 0.01). The linear regression model indicated a positive correlation between the preoperative NT/T1s ratio and the ΔC2–7 Cobb angle (R^2^ = 0.122).

**Conclusions:**

The preoperative NT/T1s ratio may be positively correlated with changes in postoperative cervical spine curvature (Cobb angle). The NT/T1s ratio may be worthy of increased attention among sagittal parameters.

## Background

In recent years, the influence of sagittal plane parameters on health-related quality of life (HRQOL) scores has attracted increasing attention from experts [1–3]. The C2–7 Cobb angle, C2–7 sagittal vertical axis (SVA) and T1 slope (T1s) are the three major sagittal parameters of the cervical spine. The C2–7 Cobb angle is a commonly used indicator for evaluating cervical curvature. Some experts have suggested that maintaining lordosis after surgery leads to a favorable clinical prognosis [4, 5]. Tang et al. [2] suggested that when the C2–7 SVA is > 40 mm, the neck disability index (NDI) score is increased. Weng et al. [6] conducted a study on patients with cervical degenerative diseases and found that because T1 is the vertebra that connects the cervical and thoracic vertebrae, its inclination plays an important role in the clinical prognosis of patients.

Maintaining the normal curvature of the cervical spine is the key to maintaining the balance of the sagittal spine. Reversal of the normal curvature of the cervical spine, including kyphosis, may occur through multiple mechanisms and may lead to mechanical pain and neurological dysfunction [7, 8]. Narrowing of the nerve foramen caused by degeneration of the intervertebral disc may cause radiculopathy, and impingement or stretching of the spinal cord, which usually occurs at the apex of the deformity, can cause symptoms of myelopathy. However, sagittal parameters have rarely been studied for cervical kyphosis patients.

Parameters related to lumbar sagittal balance and alignment, including pelvic parameters such as pelvic incidence (PI), pelvic tilt (PT) and sacral slope (SS), have recently been recognized as important parameters for assessing adult spinal deformities. The relationship among these parameters is as follows: PI = PT + SS. Zhang et al. [9] found that an increased PT/SS value may play important roles in the degeneration of lumbar scoliosis and pathogenesis of lumbar spondylolisthesis. We found a similar relationship among cervical sagittal parameters: thoracic inlet angle (TIA) = neck tilt (NT) + T1s. In previous studies, we demonstrated that the T1s is associated with clinical outcome, but the results were not specific for individuals. Therefore, we used the NT/T1s ratio as a personalized indicator. We aimed to explore the effect of the NT/T1s ratio in patients with cervical kyphosis. To our knowledge, this effect has never been reported.

## Methods

### Patient population

After approval was obtained from the Institutional Review Board, anterior cervical mixed decompression and fusion (ACHDF) patients who underwent multilevel (3 levels) cervical myelopathy during spinal surgery from January 2010 to June 2015 were retrospectively analyzed with regard to clinical and imaging examination results.

All patients were diagnosed by detailed physical examination and medical history. The inclusion criteria were as follows: (1) no spinal trauma, tumor or spinal infection; (2) preoperative, postoperative and follow-up cervical lateral radiographs; (3) no other previous cervical surgery or cervical internal fixation; (4) preoperative cervical kyphosis (C2–7 Cobb angle < 0); and (5) patients who could undergo sagittal parameter measurement (T1 vertebrae could be clearly seen on X-rays, and the sternum or sagittal ribs did not interfere with measurement of the vertebral body).

### Radiographic measurements

**Fig. 1:**
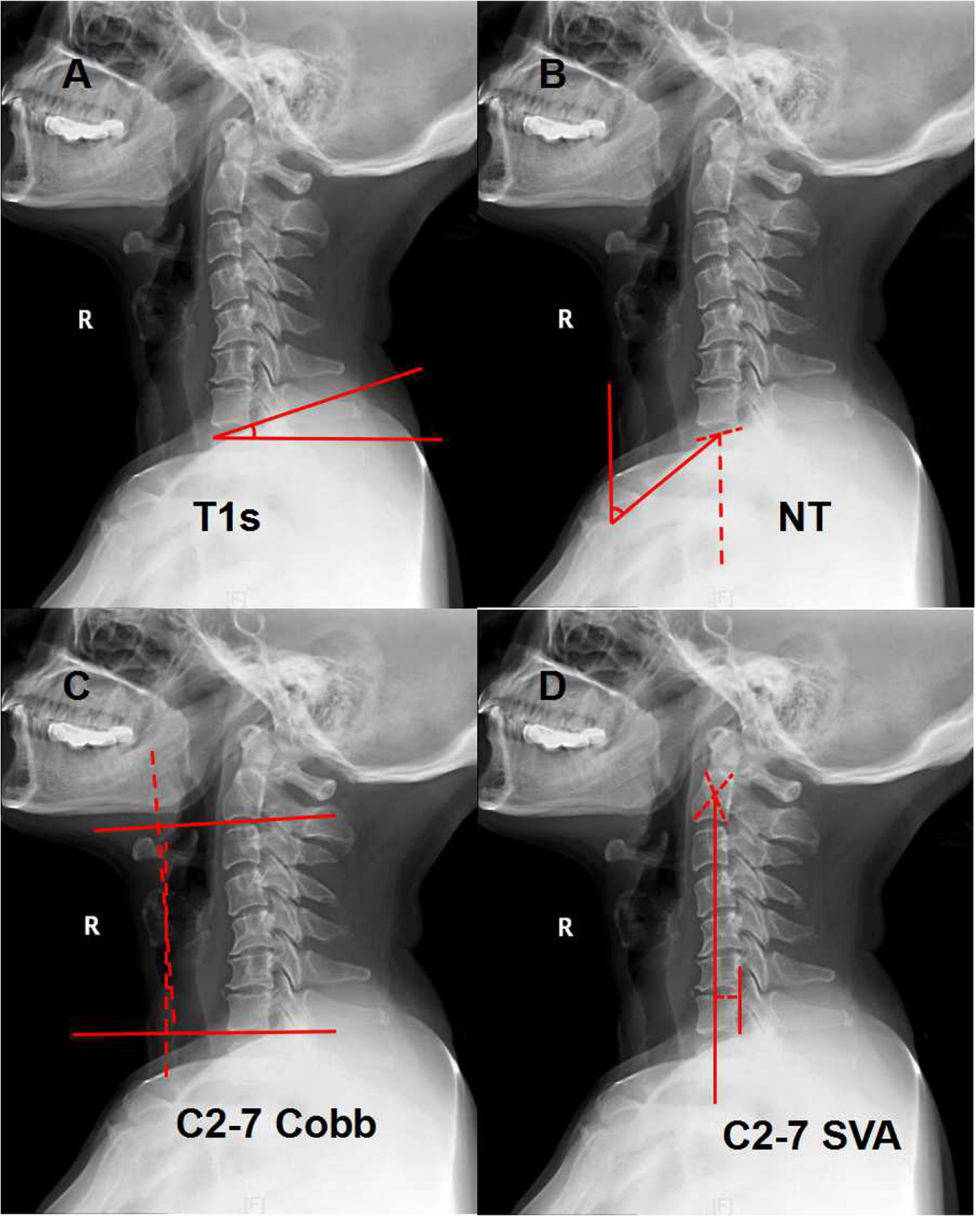
Measurements of parameters. **A**: T1s, T1 slope; **B**: NT, neck tilt; **C**: C2-7 Cobb; **D**: C2-7 SVA, sagittal vertical axis

X-ray films were obtained through a standard cervical lateral X-ray series and uploaded to our Picture Archiving and Communication System (PACS). The data were analyzed by two experienced clinicians with more than 10 years of experience. When obtaining X-rays, the patient was told to stand as upright as possible and look forward. The following parameters were measured (Fig. 1): (1) T1s [9]: the angle formed between a horizontal line and the upper end plate of T1; (2) NT [9]: the angle formed by a line connecting the upper end of the sternum and the center of the upper end plate of T1 and a line drawn from the upper end of the sternum; (3) C2–7 Cobb angle [3]: on a neutral lateral X-ray photograph, the angle formed by the line parallel to the underside of the C7 body and the line parallel to the line on the C2 body; (4) C2–7 SVA [3]: the distance between the plumb line of C2 and the posterior upper end plate of C7; positive sagittal alignment was defined as forward deviation; (5) Δvalue: the difference between the postoperative and follow-up values for each parameter.

The intragroup correlation coefficient (ICC) was used to assess interobserver reliability. The ICC was calculated using a two-way mixed model to determine the absolute consistency between observers. The internal consistency of the measurement was characterized as excellent (ICC ≥ 0.9), good (0.7 ≤ ICC < 0.9), acceptable (0.6 < ICC ≤ 0.7), poor (0.5 ≤ ICC < 0.6), or unpredictable (ICC < 0.5) [7].

### Clinical outcome measurements

The NDI score was classified as follows: 0 to 20 indicated mild dysfunction, 21 to 40 indicated moderate dysfunction, 41 to 60 indicated severe dysfunction, 61 to 80 indicated very severe dysfunction, and 81 to 100 indicated complete dysfunction or subjects with or without exaggerated symptoms who required detailed examination. Before surgery and during follow-up (at least 1 year), the clinical outcome was evaluated by the NDI and visual analog scale (VAS) scores of each patient.

### Statistical analysis

SPSS 20.0 software (IBM, New York, USA) was used for statistical processing of all data. The measurement data are expressed as‾x ± s. A paired T test was used to compare the effects of ACHDF on cervical sagittal alignment parameters and HRQOL scores. The Pearson correlation coefficient was calculated to compare the preoperative X-ray measurements and Δvalues. Linear regression analysis was used to analyze the relationship between the preoperative NT/T1 ratio and ΔCobb angle. A value of *p* < 0.05 was considered statistically significant.

## Results

### Demographic data

In total, 36 cervical kyphosis patients (male/female = 24/12) were identified; their mean age was 54.4 ± 9.7 (range, 38–78) years, and their mean BMI was 23.5 ± 2.2. The involved segments were distributed as follows: C3–6 (23 patients) and C4–7 (13 patients). All surgical segments were fused 3 months after surgery. The average follow-up time of postoperative imaging examinations and HRQOL scores was 47.2 ± 8.0 months (Table 1).

**Table 1:** General Information

Item	ACHDF
Mean age (years)	54.4 ± 9.7
BMI (kg/m2)	23.5 ± 2.2
Male	24 (66.7%)
Female	12 (33.3%)
Average follow-up (months)	47.2 ± 8.0
Surgical segment (cases)	C3-6: 23
	C4-7: 13
Surgical treatment (cases)	36

### Radiographic measurements and HRQOL scores

**Fig. 2:**
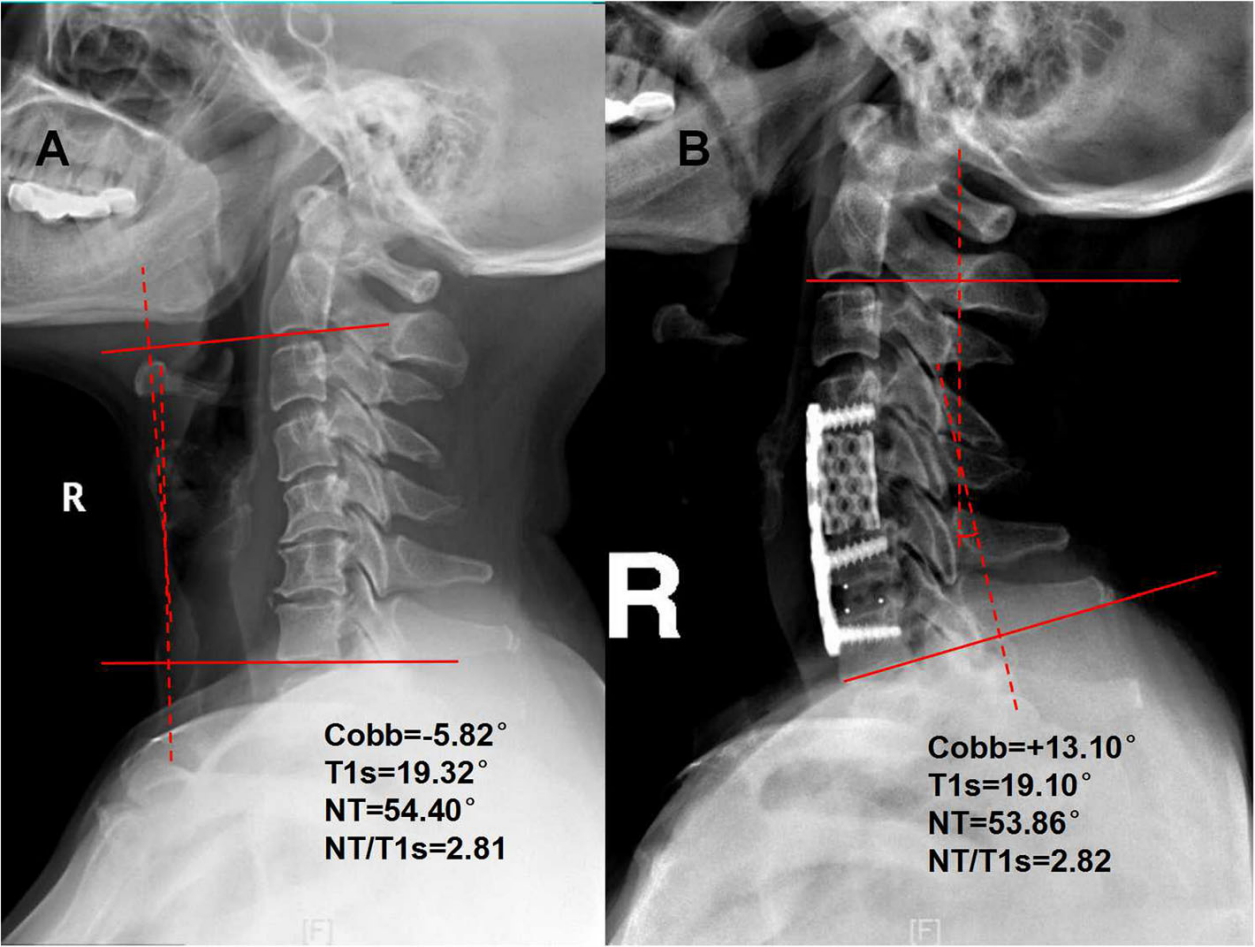
A case (preoperative and postoperative X-ray films). T1s, T1 slope; NT, neck tilt

The mean T1s was 20.73 ± 8.31° before surgery, 21.45 ± 7.74° after surgery, and 23.35 ± 5.00° at follow-up. The mean NT angle was 47.35 ± 5.00° before surgery, 45.65 ± 10.07° after surgery, and 46.08 ± 9.61° at follow-up. The mean C2–7 Cobb angle was − 5.93 ± 3.00° before surgery, 9.67 ± 6.61° after surgery, and 7.91 ± 8.73° at follow-up. The mean C2–7 SVA length was 1.41 ± 1.07 cm before surgery, 1.75 ± 0.71 cm after surgery, and 1.43 ± 1.28 cm at follow-up. Tables 2 and 3 summarize and compare the preoperative, postoperative and follow-up values of HRQOL scores and radiographic measurements. All measured radiographic variables (all ICCs > 0.7) and most other variables had good reliability (Table 2). A representative case is presented in Fig. 2.

**Table 2:** Sagittal alignment parameters, and interrater reliability for all patients

Item	Preoperative	Pre- ICC	Postoperative	Post- ICC	Follow-up	Follow-up ICC
T1s (°)	20.73 ± 8.31	0.946	21.45 ± 7.74	0.920	23.35 ± 5.00	0.873
NT (°)	47.35 ± 5.00	0.842	45.65 ± 10.07	0.962	46.08 ± 9.61	0.964
C2-7 Cobb (°)	-5.93 ± 3.00	0.796	9.67 ± 6.61	0.925	7.91 ± 8.73	0.943
C2-7 SVA (cm)	1.41 ± 1.07	0.867	1.75 ± 0.71	0.908	1.43 ± 1.28	0.782

ICC, interrater reliability; Postoperative, within 1 week after surgery; Follow-up, at least 36 months after the surgery

*T1s* T1 slope, *NT* neck tilt, *C2-7SVA* C2-7 sagittal vertical axis

**Table 3:** Cervical sagittal alignment parameters

Item	Preoperative	Postoperative	Follow-up	*p* value	p* value
T1s (°)	20.73 ± 8.31	21.45 ± 7.74	23.35 ± 5.00	0.081	0.352
NT (°)	47.35 ± 5.00	45.65 ± 10.07	46.08 ± 9.61	0.386	0.675
C2-7 Cobb (°)	-5.93 ± 3.00	9.67 ± 6.61	7.91 ± 8.73	0.000	0.022
C2-7 SVA (cm)	1.41 ± 1.07	1.75 ± 0.71	1.43 ± 1.28	0.078	0.083
VAS	6.97 ± 0.85	3.03 ± 0.70	2.92 ± 0.69	0.000	0.487
NDI	18.53 ± 3.03	10.58 ± 2.63	10.11 ± 2.18	0.000	0.409

Postoperative, within 1 week after surgery; Follow-up, at least 36 months after the surgery; *p* value, comparison between preoperative and postoperative values

*T1s* T1 slope, *NT* neck tilt, *C2-7 SVA* C2-7 sagittal vertical axis, *VAS* visual analogue scale, *NDI* neck disability index

*p value, comparison between postoperative and follow-up values

### Correlations between the sagittal alignment parameters

Pearson’s correlation coefficient was calculated to compare radiographic measurements. The preoperative NT/T1s ratio was positively correlated with the ΔC2–7 Cobb angle (*r* = 0.358, *p* < 0.05) and negatively correlated with the preoperative C2–7 Cobb angle (*r* = -0.515, *p* < 0.01) and preoperative C2–7 SVA (*r* = -0.461, *p* < 0.01). The preoperative C2–7 Cobb angle was negatively correlated with the ΔC2–7 Cobb angle (*r* = -0.337, *p* < 0.05). The preoperative C2–7 SVA was negatively correlated with the ΔC2–7 SVA (*r* = -0.510, *p* < 0.01) (Table 4).

**Table 4:** Correlation between the sagittal alignment parameters

		pre-NT/T1s	pre-C2-7 Cobb	pre-C2-7 SVA	ΔC2-7 Cobb	ΔC2-7 SVA
pre-NT/T1s	r	1	− 0.515**	− 0.461**	0.358*	0.230
	p		0.001	0.005	0.032	0.178
pre-C2-7 Cobb	r	− 0.515**	1	0.227	− 0.337*	− 0.207
	p	0.001		0.183	0.045	0.226
pre-C2-7 SVA	r	− 0.461**	0.227	1	0.187	− 0.510**
	p	0.005	0.183		0.276	0.001
ΔC2-7 Cobb	r	0.358*	− 0.337*	0.187	1	− 0.047
	p	0.032	0.045	0.276		0.784
ΔC2-7 SVA	r	0.230	− 0.207	− 0.510**	− 0.047	1
	p	0.178	0.226	0.001	0.784	

*T1s* T1 slope, *NT* neck tilt, *SVA* sagittal vertical axis

Δvalue = follow-up value minus postoperative value

^*^Correlation is significant at the *P* < 0.05 level (2-tailed)

^**^Correlation is significant at the *P* < 0.01 level (2-tailed)

**Fig. 3:**
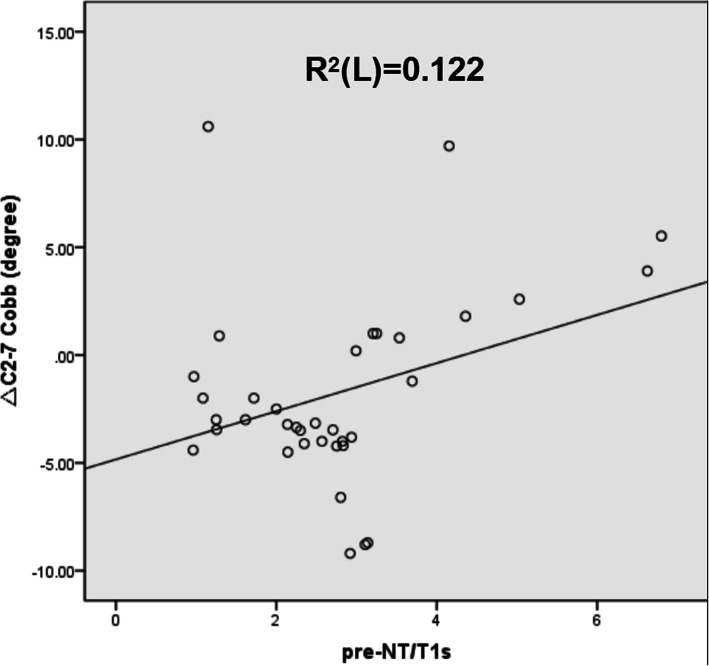
Correlations between pre-NT/T1s and ΔC2-7 Cobb. T1s, T1 slope; NT, neck tilt

The linear regression model showed the relationship between the preoperative NT/T1s ratio and the ΔC2–7 Cobb angle (Fig. 3).

## Discussion

In recent years, the influence of sagittal parameters on the clinical prognoses of patients has attracted increasing attention. Relatively few patients are diagnosed with cervical kyphosis in clinical practice, and there are few studies on the sagittal parameters of patients with kyphosis. In the lumbar spine, the following relationship is observed: PI = PT + SS. Zhang et al. [9] found that an increased PT/SS ratio may play an important role in the pathogenesis of lumbar spondylolisthesis. However, in the cervical spine, the following relationship is observed: TIA = NT + T1s. Therefore, we conducted this study to explore the role of the NT/T1s ratio in patients with cervical kyphosis.

There are several important sagittal parameters of the cervical spine. Previous studies have shown that surgical intervention to maintain C2–7 lordosis has a positive effect on the prognosis of patients, which may be due to the lower energy consumption of neck muscles and ligaments when physiological lordosis exists [10–12].

Clinically, kyphosis is often corrected, although a study by Villavicencio et al. [13] revealed that correcting kyphosis may lead to increased symptoms in patients; however, our study showed that kyphosis can be corrected to lordosis in patients, and good results can be achieved. Nonetheless, although anterior fusion surgery provides strong internal fixation support for the cervical spine, a few patients still exhibit postoperative cervical spine micromotion or even secondary kyphosis. The C2–7 SVA is an important parameter for predicting the outcome of cervical spine surgery. Tang et al. [2] confirmed that the C2–7 SVA is significantly correlated with NDI scores in patients, and the regression model predicted a C2–7 SVA threshold of 40 mm.

In asymptomatic individuals, NT is maintained at approximately 44° to minimize energy expenditure in the cervical spine [14]. T1 is affected not only by the lower cervical vertebrae but also by the upper cervical vertebrae. Lee et al. [14] demonstrated that in an asymptomatic group, when the ratio of the cervical spine tilt to the cranial tilt was 70.2 %:29.8 %, energy consumption could be minimized. Moreover, Huang et al. [15] found that an excessive T1s (> 40°) can be considered a risk factor for high energy expenditure.

A study by Lee et al. [14] revealed that NT or T1s cannot be used as a predictor if the patient is in a different posture other than standing, including a leaning, sitting, supine or prone posture, but the TIA does not change under any circumstances due to changes in body position, including the PI. The ratio of NT and T1s is used to assess horizontal gaze. When the value of T1s is smaller, the C2–7 Cobb angle changes greatly because the T1 vertebral body is equivalent to the bottom of the cervical spine, and when the T1 vertebral body is flat, the cervical spine tends to be kyphotic. In our study, the preoperative NT/T1s ratio was positively correlated with changes in postoperative cervical spine curvature. For patients with larger NT angles, the postoperative Cobb angle may be smaller and vice versa. On the basis of the results shown in Table 3, in our study, we can conclude that for patients with preoperative cervical kyphosis, surgery mainly changed the C2–7 Cobb angle, increased the degree of lordosis, and improved the prognosis. Only the C2–7 Cobb angle changed during follow-up, indicating that although fusion surgery was performed with an anterior approach, kyphosis patients still had a tendency to exhibit lordosis loss due to sagittal instability. On the basis of the results shown in Table 4, we can also conclude that the preoperative NT/T1s ratio was associated with the preoperative C2–7 Cobb angle (*r* = -0.515, *p* = 0.001); a larger NT/T1s ratio resulted in a more pronounced trend in kyphosis.

In this study, NT referred to the angle of the thoracic inlet plane, and it was almost impossible to intentionally keep the thoracic inlet plane horizontal or vertical in an upright position. The simple T1s index and the C2–7 SVA are easily affected by body position. In this study, we additionally considered the lumbosacral spine and used a new parameter, the NT/T1s ratio, to reduce interindividual differences. Compared with patients with nonposterior deformities, patients with kyphosis exhibit a larger NT/T1s ratio. It can be speculated that the NT/T1s ratio is a new indicator that may be a factor leading to the development of kyphosis, but this speculation still lacks sufficient evidence. The results of this study additionally suggest that for patients with large NT/T1s values, secondary kyphosis is likely to occur and should be prevented after surgery. This indicator may be more specific than the NT or T1s alone. Furthermore, because the T1 vertebral body is not directly affected during surgery, the NT/T1s index is fixed for a single individual and may be a good predictor of their prognosis. In addition, according to Table 4, we found that the NT/T1s ratio is correlated with the C2–7 SVA and C2–7 Cobb angle, which were previously shown to be related to the HRQOL. For patients with preoperative cervical kyphosis, we recommend that anterior fusion surgery be performed to increase the degree of lordosis to prevent subsequent lordosis loss. At the same time, the duration for which patients wear a neck brace should be extended to prevent the degree of kyphosis from increasing when the internal fixation region is not fused.

This study had several limitations. First, the relatively small sample size and retrospective nature of the analysis may have biased the results. Second, the data were collected from a single center; therefore, our results may not be generalizable to other centers. Third, the NT/T1s ratio is a good indicator, but we did not obtain a quantitative result or estimate its threshold range. Fourth, we compared the correlation between the preoperative NT/T1s ratio and the C2–7 Cobb angle. However, this is a retrospective study, and evidence for the results is lacking. We will perform prospective studies in future investigations.

## Conclusions

In patients with cervical kyphosis, the preoperative NT/T1s ratio may be positively correlated with changes in postoperative cervical spine curvature (Cobb angle). The NT/T1s ratio may be worthy of further attention.

## Data Availability

All data generated or analyzed during this study are available upon reasonable request from the corresponding author.

## Abbreviations

PT: Pelvic tilt
SS: Sacral slope
ACHDF: Anterior cervical hybrid decompression and fusion
SVA: Sagittal vertical axis
T1s: T1 slope
NDI: Neck disability index
VAS: Visual analog scale
TIA: Thoracic inlet angle
NT: Neck tilt
HRQOL: Health-related quality of life
PI: Pelvic incidence
PACS: Picture Archiving and Communication System
ICC: Intragroup correlation coefficient

## References

[1] Kim TH, Lee SY, Kim YC, Park MS, Kim SW (2013). T1 slope as a predictor of kyphotic alignment change after laminoplasty in patients with cervical myelopathy. Spine (Phila Pa 1976).

[2] Tang JA, Scheer JK, Smith JS, Deviren V (2015). The impact of standing regional cervical sagittal alignment on outcomes in posterior cervical fusion surgery. Neurosurgery.

[3] Hyun SJ, Kim KJ, Jahng TA, Kim HJ (2016). Relationship between T1 slope and cervical alignment following multilevel posterior cervical fusion surgery: impact of T1 slope minus cervical lordosis. Spine (Phila Pa 1976).

[4] Katsuura A, Hukuda S, Saruhashi Y, Mori K (2001). Kyphotic malalignment after anterior cervical fusion is one of the factors promoting the degenerative process in adjacent intervertebral levels. Eur Spine J.

[5] Grosso MJ, Hwang R, Mroz T, Benzel E, Steinmetz MP (2013). Relationship between degree of focal kyphosis correction and neurological outcomes for patients undergoing cervical deformity correction surgery. J Neurosurg Spine.

[6] Weng C, Wang J, Tuchman A, Wang J (2016). Influence of T1 slope on the cervical sagittal balance in degenerative cervical spine: an analysis using kinematic MRI. Spine (Phila Pa 1976).

[7] Ferch RD, Shad A, Cadoux-Hudson TA, Teddy PJ (2004). Anterior correction of cervical kyphotic deformity: effects on myelopathy, neck pain, and sagittal alignment. J Neurosurg.

[8] Baptiste DC, Fehlings MG (2006). Pathophysiology of cervical myelopathy. Spine J.

[9] Zhang J, Hai Y, Yang J, Pan A, Zhang Y. Increased PT/SS may play an important role in the pathogenesis of lumbar spondylolisthesis with degenerative lumbar scoliosis. Clin Neurol Neurosurg. 2018;166:23–30. 10.1016/j.clineuro.2018.01.018 29358108

[10] Jenkins LA, Capen DA, Zigler JE, Nelson RW, Nagelberg S. Cervical spine fusions for trauma. A long-term radiographic and clinical evaluation. Orthop Rev. 1994;(Suppl):13–19. 7854834

[11] Kawakami M, Tamaki T, Yoshida M, Hayashi N, Ando M, Yamada H (1999). Axial symptoms and cervical alignments after cervical anterior spinal fusion for patients with cervical myelopathy. J Spinal Disord.

[12] Park MS, Kelly MP, Lee DH, Min WK, Rahman RK, Riew KD (2014). Sagittal alignment as a predictor of clinical adjacent segment pathology requiring surgery after anterior cervical arthrodesis. Spine J.

[13] Villavicencio AT, Babuska JM, Ashton A, Busch E (2011). Prospective, randomized, double-blind clinical study evaluating the correlation of clinical outcomes and cervical sagittal alignment. Neurosurgery.

[14] Lee SH, Kim KT, Seo EM, Suk KS, Kwack YH, Son ES. The influence of thoracic inlet alignment on the craniocervical sagittal balance in asymptomatic adults. J Spinal Disord Tech. 2012;25(2):E41-7. 10.1097/BSD.0b013e3182396301 22037167

[15] Huang Y, Lan Z, Xu W (2019). Analysis of sagittal alignment parameters following anterior cervical hybrid decompression and fusion of multilevel cervical Spondylotic myelopathy. BMC Musculoskelet Disord.

